# Carbon, nitrogen and phosphorus contents and their ecological stoichiometric characteristics in leaf litter from the Jianfengling Tropical Montane Rainforest

**DOI:** 10.3389/fpls.2024.1478094

**Published:** 2024-09-24

**Authors:** Shuxuan Yin

**Affiliations:** Department of Environmental Science, Inner Mongolia University, Hohhot, China

**Keywords:** leaf litter, organic carbon, total nitrogen, total phosphorus, stoichiometry

## Abstract

Investigating carbon (C), nitrogen (N) and phosphorus (P) contents and ecological stoichiometric characteristics in leaf litter from tropical rainforests is crucial for elucidating nutrient cycling and energy flow in forest ecosystems. In this study, a 60-ha tropical montane rainforest dynamic monitoring plot in Jianfengling, was selected as the research site and 60 subplots were selected for detailed study. Leaf litter was collected monthly throughout 2016, branches of similar height were placed atthe four corners of each sample square to support a nylon cloth (1 m× 1 m) with 1 mm apertures. The collected plant leaves were sorted,placed into envelopes, labelled, and transported to the laboratory and samples from various plant species were identified, resulting in a total of 107 samples collected and analyzed. For the 31 dominant species, the leaf litter had C, N and P contents of 312.71 ± 28.42, 4.95 ± 0.46 and 0.40 ± 0.03 g/kg, respectively. The C:N, C:P and N:P ratios were 63.61 ± 7.50, 790.91 ± 82.30 and 12.49 ± 1.00, respectively, indicating moderate variability. The C, N and P contents exhibited greater variability among the plant groups, indicating significant heterogeneity among the samples. In contrast, the data from the subplots exhibited less variability, highlighting significant homogeneity. Overall, the mean carbon, nitrogen and phosphorus contents in the leaf litter from tropical montane rainforests were lower than those observed at national and global scales. The N:P ratios in leaf litter below 14 indicated that nitrogen limited litter decomposition in Jianfengling. However, no significant correlations were observed between the C, N and P contents and their stoichiometric ratios in leaf litter and those in soil. The above results provide important reference data and scientific basis for the nutrient cycling and energy flow processes, and in the future, we can explore the limiting role and mechanism of nitrogen in the decomposition process of leaf litter.

## 1 Introduction

Ecological stoichiometry, a popular field of ecological research in recent years, focuses on the balance between various interacting chemical elements in ecosystems (Elser et al., 2000; Peguero et al., 2023). Plants require over 30 elements for growth, with carbon (C), nitrogen (N) and phosphorus (P) being crucial for constructing plant structures and supporting metabolic processes. Carbon, the most dominant element in plants, comprises about 50% of their dry weight. Additionally, carbon is vital for producing sugars necessary for plant growth, reproduction, and structural development (Friedlingstein et al., 2020; Cook-Patton et al., 2020). Nitrogen is crucial for synthesizing all enzymes and chlorophyll in plants and plays a key role in the primary production of organic compounds. Phosphorus is vital for controlling ribosome synthesis in plants and is a key component of RNA, DNA and ATP. Moreover, phosphorus significantly influences information transfer, energy storage, and cellular architecture in plants (Farrer and Goldberg, 2009; Du et al., 2020; Sardans et al., 2012). To analyze organisms as integrated systems and connect research across different areas, ecologists have proposed linking the molecular structure of genes to various levels of ecosystem organization and dynamics. This approach forms the basis of ecological stoichiometry, which provides new insights into leaf-litterfall-soil nutrient cycling and regulation and is a key indicator for maintaining and preserving forest communities. Elser et al. (2000) indicated that the stoichiometry of C, N and P in organisms is closely associated with their growth. Furthermore, autotrophic organisms exhibit variable C:N:P ratios both within and among species. These ratios in plant leaves influence plant growth, nutrient cycling, and primary production (Falcell and Pickett, 1991; Tie et al., 2023). Therefore, investigating the carbon, nitrogen and phosphorus contents in plants and their stoichiometric characteristics is crucial for elucidating their dynamic role in regional biogeochemical cycles.

Litterfall consists of all organic matter produced by the above-ground parts of plants and deposited onto the surface, including components such as leaf litter, propagules and twigs (Tian et al., 2024). Litter serves as a crucial link between plants and soil and plays a key role in nutrient cycling and energy flow within forest ecosystems (Njoroge et al., 2022; Zhang et al., 2023a; Li et al., 2023). Notably, leaf litter decomposes rapidly and has been extensively investigated by scholars. Yuan et al. (2024) quantified global patterns of C, N and P contents in litter and its returns. The study identified climate and soil as the main factors influencing litter ecological stoichiometry. Additionally, C, N and P contents in leaf litter were influenced by mycorrhizal associations, taxonomic divisions and/or life forms. He et al. (2024) investigated the dynamics of decomposition and elemental release from both single and mixed types of litter, their non-additive effects on soil C:N:P ratios and the potential factors regulating these non-overlapping effects. The results revealed that mixing different types of leaves promoted mass loss and elemental release, while single types of leaf litter exhibited greater residual mass. The effects of decomposing mixed leaf litter on residual mass, elemental release and soil C:N:P stoichiometry varied based on the litterfall type and showed non-additive effects. This indicates that carbon, nitrogen and phosphorus contents in plant leaves and their stoichiometric characteristics are crucial indicators of soil nutrients. Plant functional traits can respond to changes in the living environment and have an impact on ecosystem processes and functions. Functional traits reflect various aspects of a species’ use of natural resources such as light energy and soil nutrients, as well as its life history. At the small scale, elevation and relief are the two most critical topographic factors affecting plant functional traits in tropical broadleaf evergreen forests, while soil water content and total nitrogen content are the most important soil factors affecting plant functional traits in subtropical broadleaf evergreen forests (Ke et al., 2014). Therefore, extensive research on these issues is needed in the tropical regions.

Jianfengling is a unique tropical montane rainforest and semi-deciduous monsoon rainforest located in the western part of Hainan Island (Jun-pei and Qi-han, 1988). Numerous studies have investigated the structure and diversity of vegetation communities and the physicochemical properties of soils in this region. However, few studies have examined the eco-chemometrics of carbon, nitrogen and phosphorus in the leaf litter from tropical montane rainforests in Jianfengling. The Jianfengling tropical montane rainforest, a transitional ecosystem between tropical and subtropical rainforests, is a key site for investigating both types of rainforests globally (Jing-Yun et al., 2004). Therefore, analyzing the stoichiometric characteristics of carbon, nitrogen and phosphorus in the leaf litter from the Jianfengling tropical rainforest is crucial for improving and supporting existing forest data. In this study, a 60-ha dynamic monitoring plot in the Jianfengling tropical montane rainforest was selected as the research site. Litterfall was collected monthly throughout 2016, and the species of the leaf litter were identified. The carbon, nitrogen and phosphorus contents in various leaf litter types were analyzed to investigate the C, N and P contents and the C:N, C:P and N:P ratios in dominant plant species and different subplots of the tropical montane rainforest in Jianfengling. To thoroughly elucidate the stoichiometric characteristics of the ‘leaf-litterfall-soil’ system, we analyzed data on the relationships between leaf litter properties and soil physical and chemical properties across 60 subplots. Based on the phylogenetic signals of carbon, nitrogen, phosphorus and stoichiometric characteristics and ecological traits of the litter leaf in the tropical montane rainforest region of the Jianfengling, we explored which stoichiometric characteristics are the main limiting factors affecting forest function and ecosystem material cycling, with a deeper discussion on whether there are any correlation between litter decomposition and soil nutrients, and explored potential correlations of plant functional traits by observing the developmental signals of ecological traits.

## 2 Materials and methods

### 2.1 Study area

Hainan Jianfengling National Nature Reserve is positioned at the boundary of Ledong County and Dongfang City in the southwestern part of Hainan Island (18°20′–18°57′N, 108°41′–109°12′E). This nature reserve is one of the few tropical forest ecosystems in China, covering a total area of about 640 km^2^ (Xu et al., 2015). Additionally, the reserve exhibits a tropical island monsoon climate, characterized by indistinguishable seasons and high levels of precipitation (Xu et al., 2015). The highest peak in the reserve rises over 1,400 m above sea level, while the lowest elevation is about 200 m. Owing to its significant altitude difference and complex topography, the reserve serves as a vast gene pool, containing over 2,800 vascular plants and more than 300 tree species. The reserve includes nearly all vegetation types found in tropical regions. Among these vegetation types, tropical montane rainforest has the highest distribution area in the Jianfengling forest (Zhongmin et al., 1998). The Jianfengling 60-ha forest biodiversity dynamic monitoring plot (1000 m east-west, 600 m north-south), known as the ‘Jianfengling large plot’, is located in the primary forest of one of the five sub-areas within the Jianfengling tropical mountain rainforest forest region. This plot, established from 2009 to 2012, adheres to the standards set by the Tropical Forest Research Centre of the Smithsonian Tropical Research Institute in the United States (Xu et al., 2015).

### 2.2 Sample collection and analysis

In the 60-ha (1000 m × 600 m) dynamic monitoring plots in Jianfengling, 60 subplots were selected for detailed study (Liu et al., 2021). To collect leaf litter, branches of similar height were placed at the four corners of each sample square to support a nylon cloth (1 m × 1 m) with 1 mm apertures. The collected plant leaves were sorted, placed into envelopes, labelled, and transported to the laboratory. In the lab, the leaves were dried at 65 ° for 24 h in an oven. After drying, the plant leaves were weighed, identified by experts, and classified into a total of 107 leaf litter species. Leaf litter of the same species was then aggregated for analysis. According to Xu et al. (2015) study on species composition and dominance in the Jianfengling region, this study analyzed the carbon, nitrogen and phosphorus contents from 31 dominant species in this region and their stoichiometric characteristics, as well as the phylogenetic signals of ecological traits.

### 2.3 Analytical test methods and data processing

Microsoft Office Excel 2021 software was used to calculate and organize each sample data. IBM SPSS Statistics 27 software was used to analyze and process the data for determining the stoichiometric characteristics of carbon, nitrogen, phosphorus and other elements in the leaf litter samples. Soil physicochemical property data were obtained from Liu et al. (2021). The stoichiometric characteristics of leaf litter, including the C:N, C:P and N:P ratios and soil physicochemical properties across 60 sample plots in the Jianfengling area, were analyzed. Correlation analyses were performed using Corrplot to explore potential correlations between leaf litter decomposition and soil physicochemical properties. Statistical significance was set at a p-value of <0.05. Additionally, the carbon, nitrogen and phosphorus contents of leaf litter from 31 dominant plant species and 60 subplots were statistically analyzed using boxplots. This analysis examined the correlation between the dominant plant species and the sample plots and observed the degree of data dispersion. A smaller p-value indicated a stronger significant difference. Hierarchical Clustering Analysis (HCA) was used to group the 60 subplots into microhabitats based on altitude, convexity, and slope, in order to analyze the phylogenetic signals of ecological characteristics across all studied species. In this dendrogram, each leaf node represents a sample or data point, and the inner nodes represent the clustering results. The branch length of the dendrogram indicates the similarity and distance between samples or data points, with shorter branch lengths indicating higher similarity.

## 3 Results and analysis

### 3.1 C, N and P contents and stoichiometric ratios across 60 subplots

**Table 1** presents the statistical results for C, N, P contents and C:N, C:P and N:P ratios in leaf litter from the tropical montane rainforest in Jianfengling. The C, N and P contents in the leaf litter moderately varied from 242.29 to 378.27, 4.01 to 6.14 and 0.29 to 0.48 g/kg, respectively. The corresponding C:N, C:P and N:P ratios ranged from 45.82 to 80.21, 613.06 to 1012.75 and 10.49 to 15.44, respectively. The leaf litter in tropical montane rainforests exhibited mean C, N and P contents of 312.71 ± 28.42, 4.95 ± 0.46 and 0.40 ± 0.03 g/kg, respectively. The mean C: N, C: P and N: P ratios were 63.61 ± 7.50, 790.91 ± 82.30 and 12.49 ± 1.00, respectively.

**Table 1:** Statistical results of C, N and P contents and C: N, C: P and N: P ratios (n = 60).

	Min	Max	Mean	SD	SEM	CV %
TOC g/kg	242.29	378.27	312.71	28.42	3.64	9.09
TN g/kg	4.01	6.14	4.95	0.46	0.06	9.35
TP g/kg	0.29	0.48	0.40	0.03	0.00	8.29
C: N	45.82	80.21	63.61	7.50	0.96	11.79
C: P	613.06	1012.75	790.91	82.30	10.54	10.41
N: P	10.49	15.44	12.49	1.00	0.13	8.01

### 3.2 C, N and P contents in leaf litter across different plant species and subplots

The organic carbon, total nitrogen and total phosphorus (TP) contents and different species in leaf litter from 31 dominant plant species in the sample area were summarized and analyzed (**Table 2**). The organic carbon content in leaf litter ranged from 191.57 to 494.20 g/kg, with an average of 323.43 ± 87.67 g/kg and a coefficient of variation (CV) of 27.11%. *Cryptocarya chinensis* exhibited the highest average organic carbon content at 473.68 ± 13.63 g/kg and a lower CV of 2.88%. However, *Neolitsea phanerophlebia* had the lowest average organic carbon content at 191.57 ± 17.99 g/kg, with a higher CV of 14.10%.

**Table 2:** Carbon, nitrogen, and phosphorus content of different plant dominant species and sun or shade-loving species as well as nitrogen-fixing (NF) and non-nitrogen-fixing (NNF) types (n=31).

Dominant species	C (g/kg)	N (g/kg)	P (g/kg)	Species	Types
Altingia obovata	394.51± 3.88	8.35± 0.87	0.86± 0.20	shade	NNF
Beilschmiedia	415.64± 14.52	10.53± 0.29	0.44± 0.04		
Beilschmiedia brevipaniculata	294.35± 20.95	2.91± 0.61	0.29± 0.05	sun	NF
Beilschmiedia glauca	432.88± 10.00	13.75± 0.96	0.30± 0.03	sun	NF
Beilschmiedia tungfangensis	325.28± 23.15	3.65± 0.77	0.39± 0.07	sun	NF
Canarium album	365.38± 7.29	10.97± 0.23	0.66± 0.11	sun	NNF
Fagaceae	360.61± 105.26	5.45± 2.93	0.27± 0.08		
Castanopsis	429.82± 16.87	13.8± 0.27	0.58± 0.10		
Castanopsis carlesii	432.95± 34.08	6.80± 4.63	0.35± 0.14	shade	NF
Castanopsis concinna	449.78± 32.01	7.70± 1.62	0.44± 0.08	sun	NF
Castanopsis hystrix	335.24± 23.86	8.48± 1.79	0.38± 0.07	shade	NF
Castanopsis jianfenglingensis	448.28± 31.74	7.32± 2.22	0.41± 0.05	shade	NF
Castanopsis tonkinensis	291.1± 20.72	6.31± 1.33	0.43± 0.08	shade	NF
Cyclobalanopsis patelliformis	440.27± 0.56	12.21± 0.14	0.86± 0.20	sun	NNF
Lithocarpus amygdalifolius	456.39± 10.4	12.41± 0.91	0.15± 0.02	shade	NNF
Lithocarpus fenzelianus	435.31± 32.68	10.37± 0.54	0.21± 0.05	shade	NNF
Gironniera subaequalis	397.56± 14.49	17.23± 0.73	0.38± 0.01	sun	NNF
Ilex goshiensis	356.63± 45.34	8.26± 7.67	0.34± 0.03		
Lauraceae	395.65± 13.00	10.58± 0.38	0.37± 0.14		
Cinnamomum validinerve	402.31± 22.65	4.93± 0.56	0.41± 0.02	shade	NNF
Cryptocarya chinensis	473.68± 22.32	13.39± 0.62	0.24± 0.07	shade	NNF
Cryptocarya metcalfiana	326.78± 23.26	5.34± 1.12	0.41± 0.08	shade	NNF
Neolitsea phanerophlebia	191.57± 13.63	3.32± 0.70	0.28± 0.05	shade	NNF
Litsea baviensis	294.53± 20.96	5.83± 1.23	0.74± 0.14	sun	NNF
Alseodaphne hainanensis	400.04± 4.76	10.54± 0.10	0.52± 0.09	sun	NNF
Livistona saribus	399.69± 4.53	8.37± 1.46	1.01± 0.29	sun	NNF
Madhuca hainanensis	383.12± 4.52	2.33± 0.54	0.30± 0.00	sun	NF
Prismatomeris tetrandra	319.3± 74.74	8.51± 7.75	0.45± 0.01	sun	NNF
Wendlandia uvariifolia	297.92± 135.76	6.78± 6.34	0.51± 0.12	shade	NNF
Winchia calophylla	407.93± 1.01	12.07± 0.20	0.17± 0.01	sun	NNF
Xanthophyllum hainanense	408.9± 10.74	14.34± 2.31	0.63± 0.10	sun	NNF

The total nitrogen content in lead litter ranged from 1.94 to 17.71 g/kg, with an average of 4.82 ± 1.76 g/kg and a CV of 36.45%. *Gironniera subaequalis* exhibited the highest total nitrogen content at 17.23 ± 0.42 g/kg, with a CV of 4.23%. In contrast, *Madhuca hainanensis* had the lowest average organic carbon content at 2.33 ± 0.38 g/kg, with a CV of 23.28%.

The TP content in leaf litter ranged from 0.14 to 1.34 g/kg, with a mean of 0.42 ± 0.10 g/kg and a CV of 23.62%. *Livistona saribus* exhibited the highest mean TP content at 1.01 ± 0.17 g/kg, with a CV of 28.56%. Conversely, *Lithocarpus amygdalifolius* had the lowest mean TP content at 0.15 ± 0.01 g/kg, with a CV of 10.24%.

The sun-loving and shade-loving plant species leaf litter had C, N and P contents of 326.18 ± 90.34, 5.25 ± 1.98 and 0.45 ± 0.12 g/kg and 322.62 ± 105.16, 4.79 ± 1.61 and 0.39 ± 0.07 g/kg. There was no significant difference between the two fractions as the p-value >0.05.

The p-value of organic carbon between the nitrogen-fixing and non-nitrogen-fixing types was 0.02, which exists a significant difference. Their C, N and P contents of 393.07 ± 62.21, 5.45 ± 1.99 and 0.42 ± 0.07 g/kg and 288.25 ± 90.89, 4.82 ± 1.71 and 0.43 ± 0.42 g/kg.

**Figure 1:**
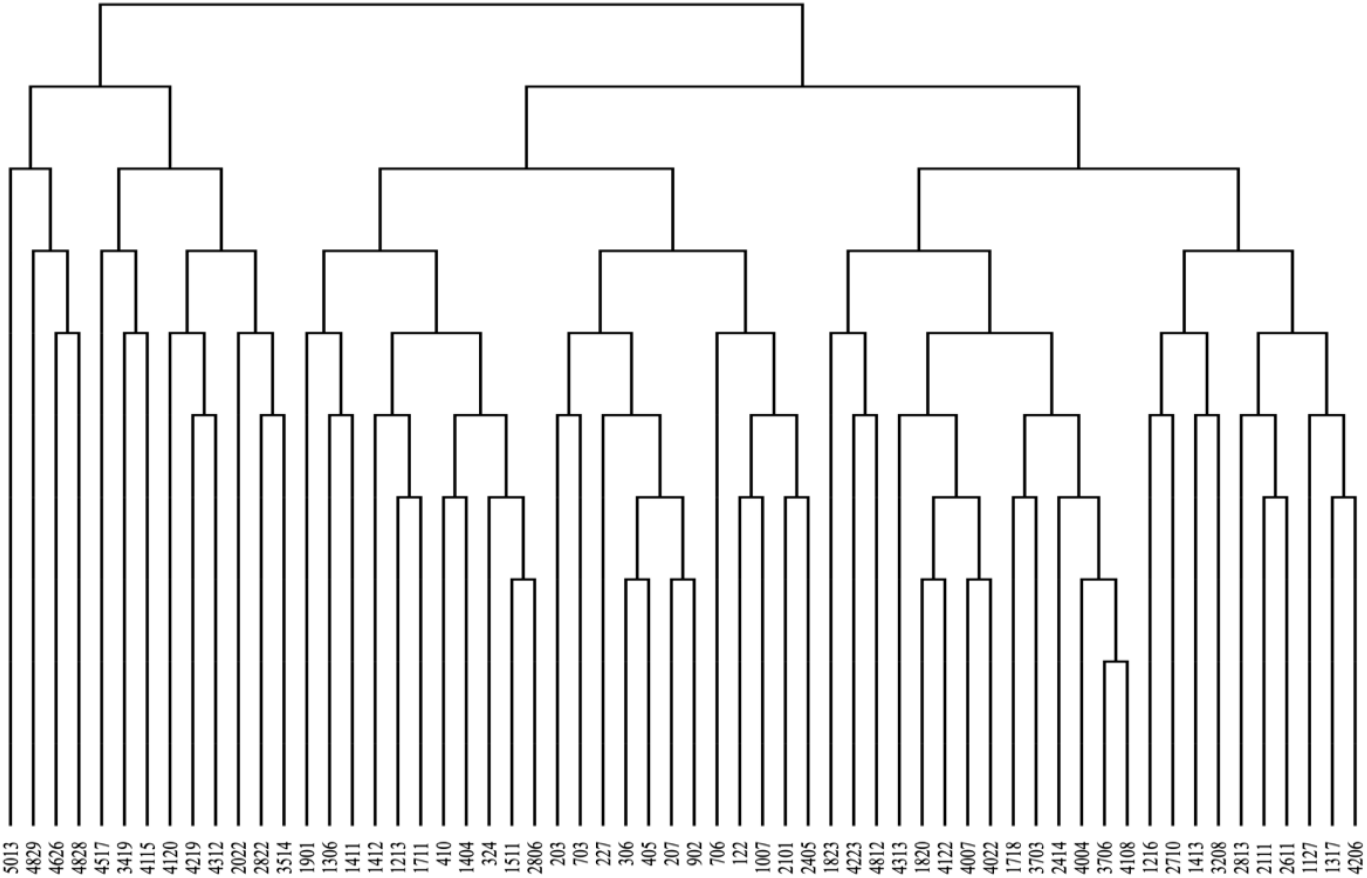
Microhabitat classification of 60 subplots based on altitude, convexity, and slope.

Based on altitude, convexity, and slope, the 60 sample plots were divided into three categories (**Figure 1**): numbered 5013-3514, 1901-2405, and 1823-4206, respectively; Plot 1 had an altitude of 966.46 - 1010.54, a convexity of -0.58 - 4.67, and a slope of 16.64 - 45.58; Sample plot 2 elevation is 870.47 - 909.52, convexity is -4.00 - 3.88, slope is 5.61 - 30.01; Sample plot 3 elevation is 917.35 - 961.58, convexity is -5.33 - 7.19, slope is 14.66 - 31.56.

Four different types of plants, sun or shade-loving species as well as NF and NNF types were selected from the subplots (**Table 3**). The effect of phylogenetic signals of ecological characteristics and altitude, convexity, and slope on plant functional trait was analyzed by examining the ratio of dry weight to total dry weight of leaf litter in the three fractions. *Beilschmiedia brevipaniculata* and *Castanopsis carlesii* are sun-loving and NF and shade-loving and NF types, respectively, and their leaf litter content decreased and then increased with altitude; *Gironniera subaequalis* is the sun-loving and NNF type, and its leaf litter content gradually decreased with increasing altitude; *Altingia obovate* is a shade-loving and NNF type, and its leaf litter content decreased significantly with altitude. In addition, *Gironniera subaequalis* and *Altingia obovate* decreased in leaf litter content as the slope steepened. And the relationship between them and convexity is not obvious.

**Table 3:** The ratio of dry weight to total dry weight of leaf litter for the four representative plants in the three fractions.

Representative plant	Plot 1	Plot 2	Plot 3	Species	Types
Beilschmiedia brevipaniculata %	4.54	3.04	0.86	sun	NF
Castanopsis carlesii %	3.51	4.12	2.56	shade	NF
Gironniera subaequalis %	3.57	11.76	5.78	sun	NNF
Altingia obovate %	3.27	11.91	3.45	shade	NNF

**Figure 2:**
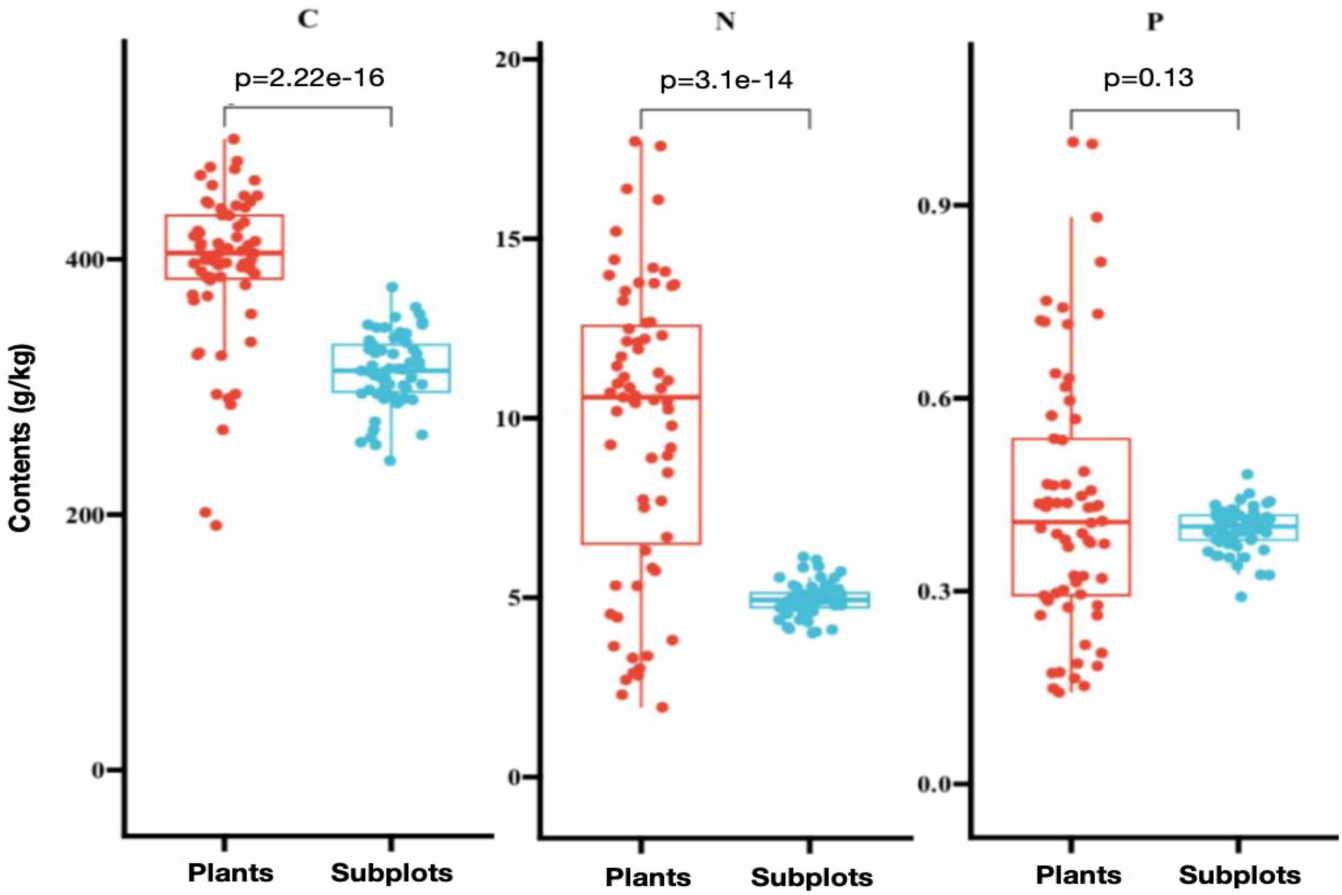
Correlations between carbon, nitrogen and phosphorus components across 60 subplots and 31 dominated plant species. The values at the top of the graph indicate the *p*-values for the correlations between plant species and sample plots.

No significant differences in C, N and P contents were observed across 60 subplots and 31 dominated plant species (**Figure 2**). Additionally, the C, N and P contents in the plant taxa exhibited higher variability and more outliers, indicating significant heterogeneity in the samples. In contrast, the data from the subplots exhibited less variability, indicating distinct homogeneity.

### 3.3 Correlations between leaf litter and soil C, N and P contents and their stoichiometric ratios

**Figure 3:**
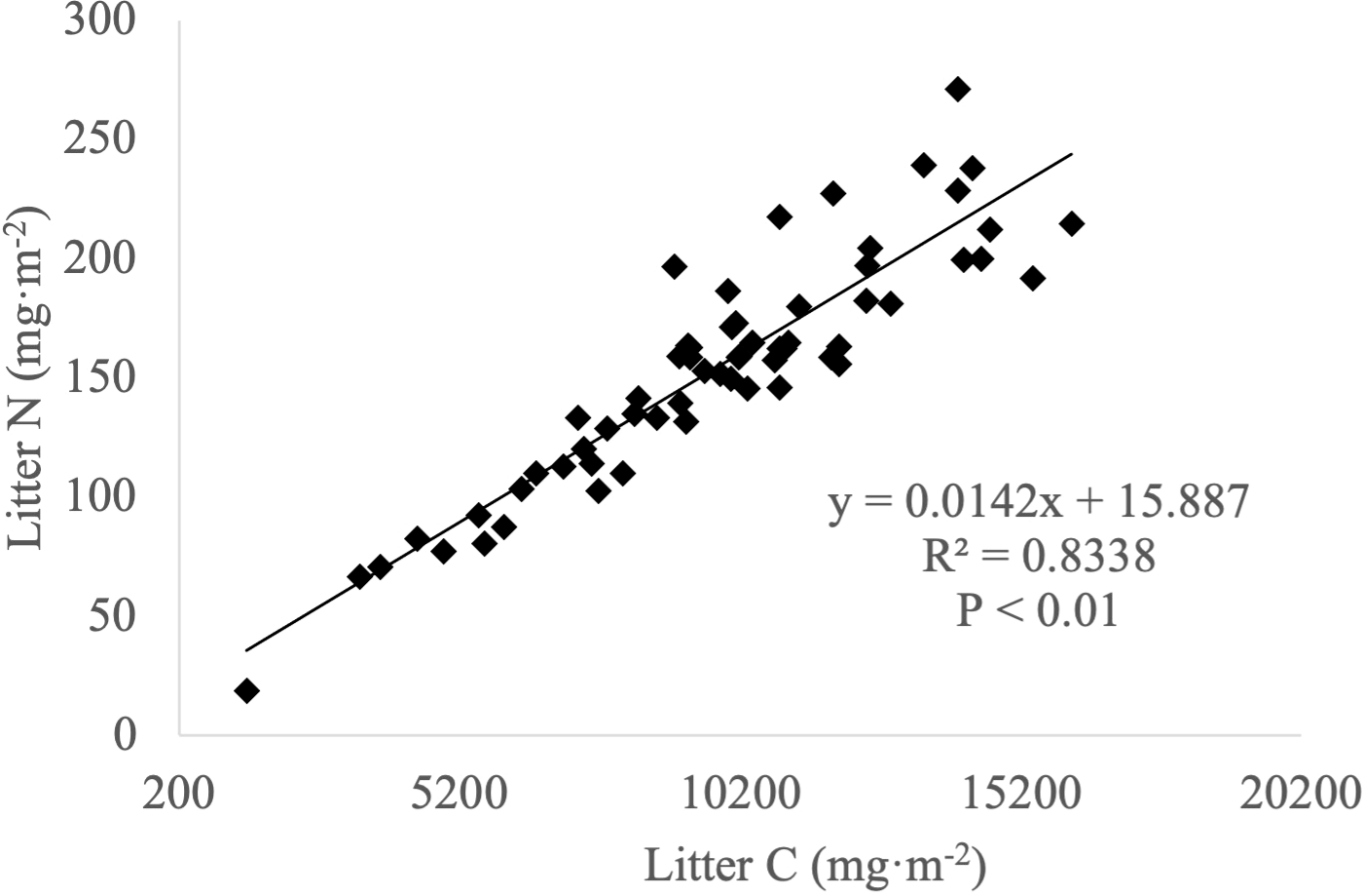
Relationship between carbon and nitrogen contents in leaf litter.

**Figure 4:**
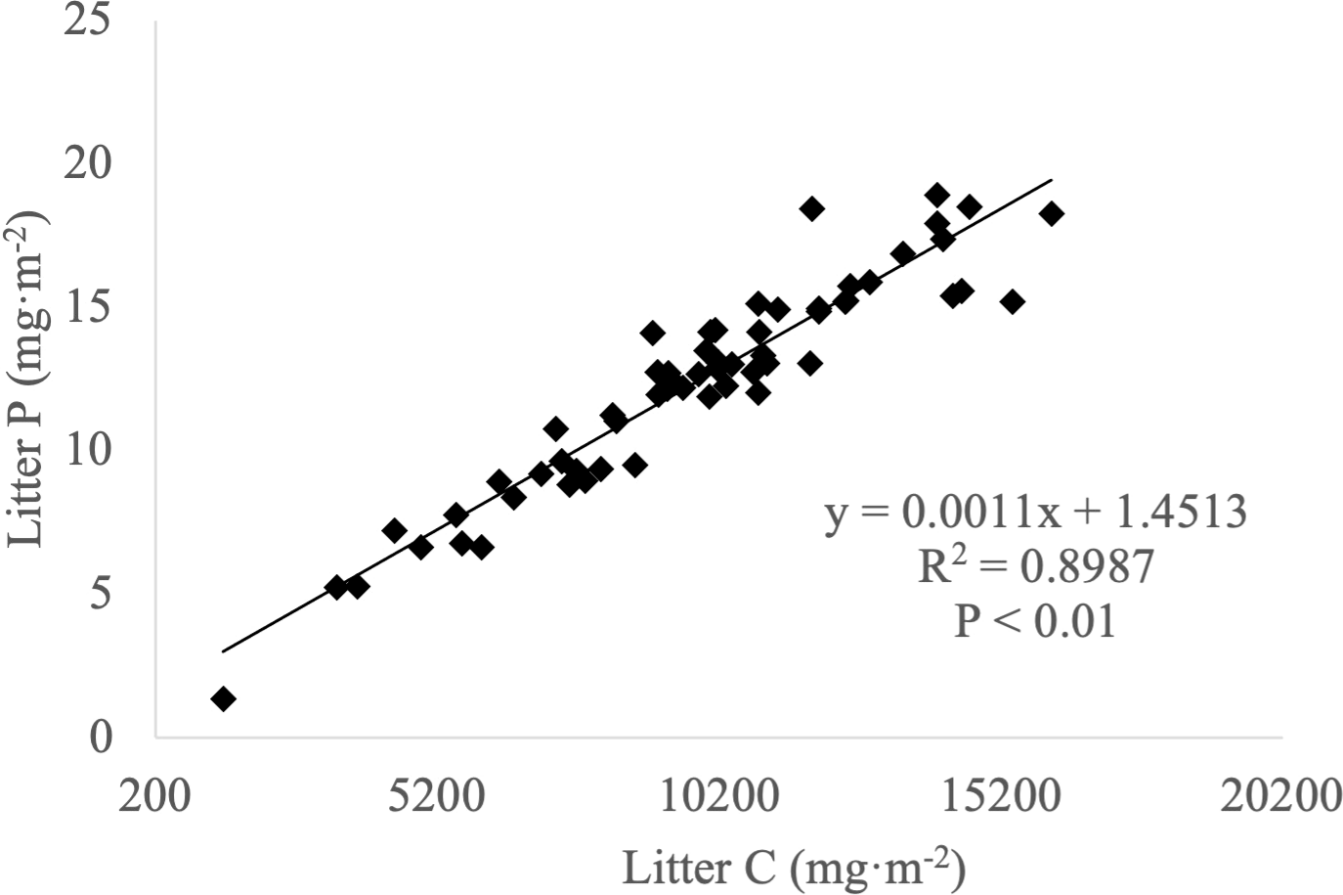
Correlation between carbon and phosphorus contents in leaf litter.

**Figure 5:**
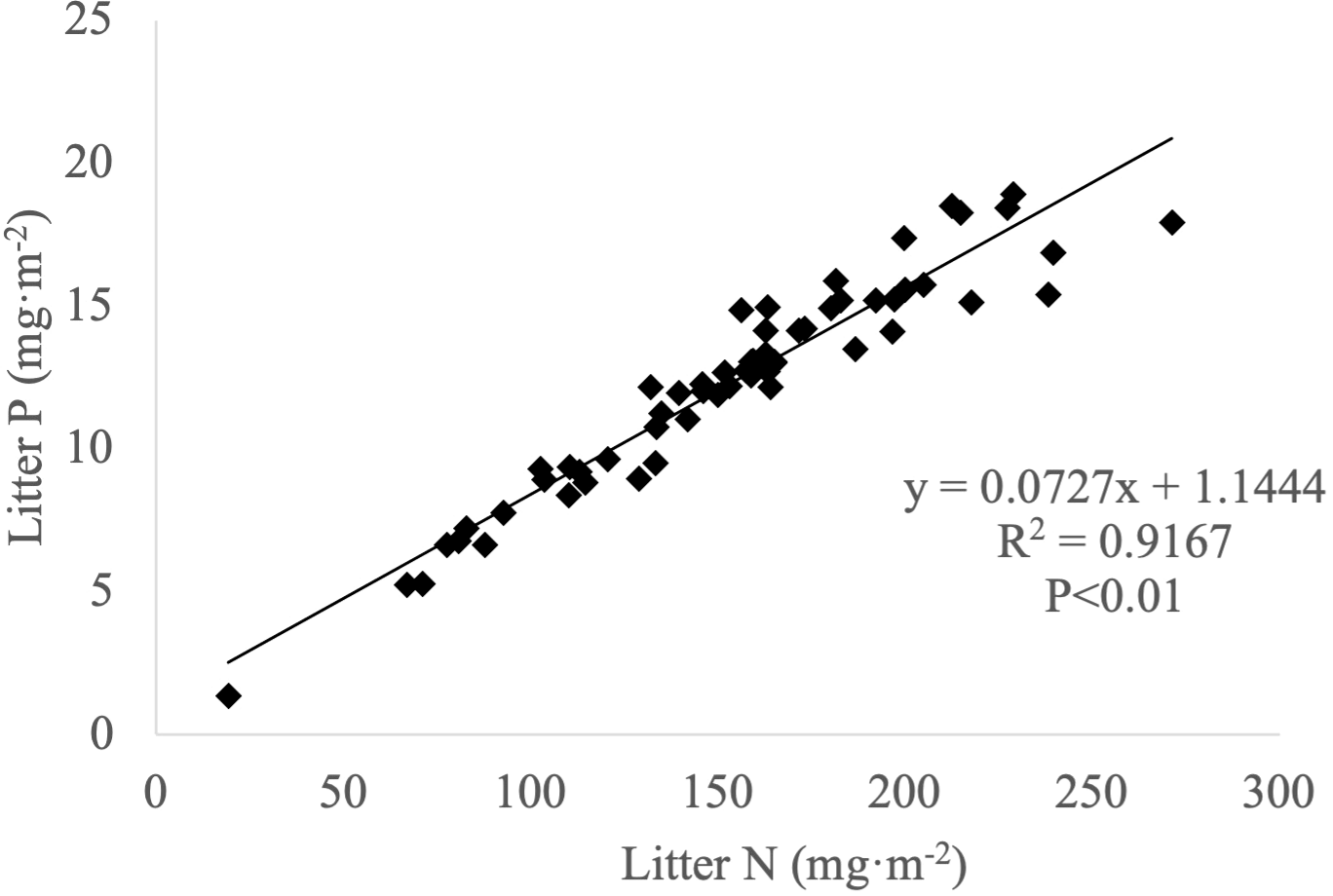
Correlation between nitrogen and phosphorus contents in leaf litter.

Significant positive correlations were observed between carbon and nitrogen (**Figure 3**), carbon and phosphorus (**Figure 4**) and nitrogen and phosphorus (**Figure 5**) contents in the leaf litter.

**Figure 6:**
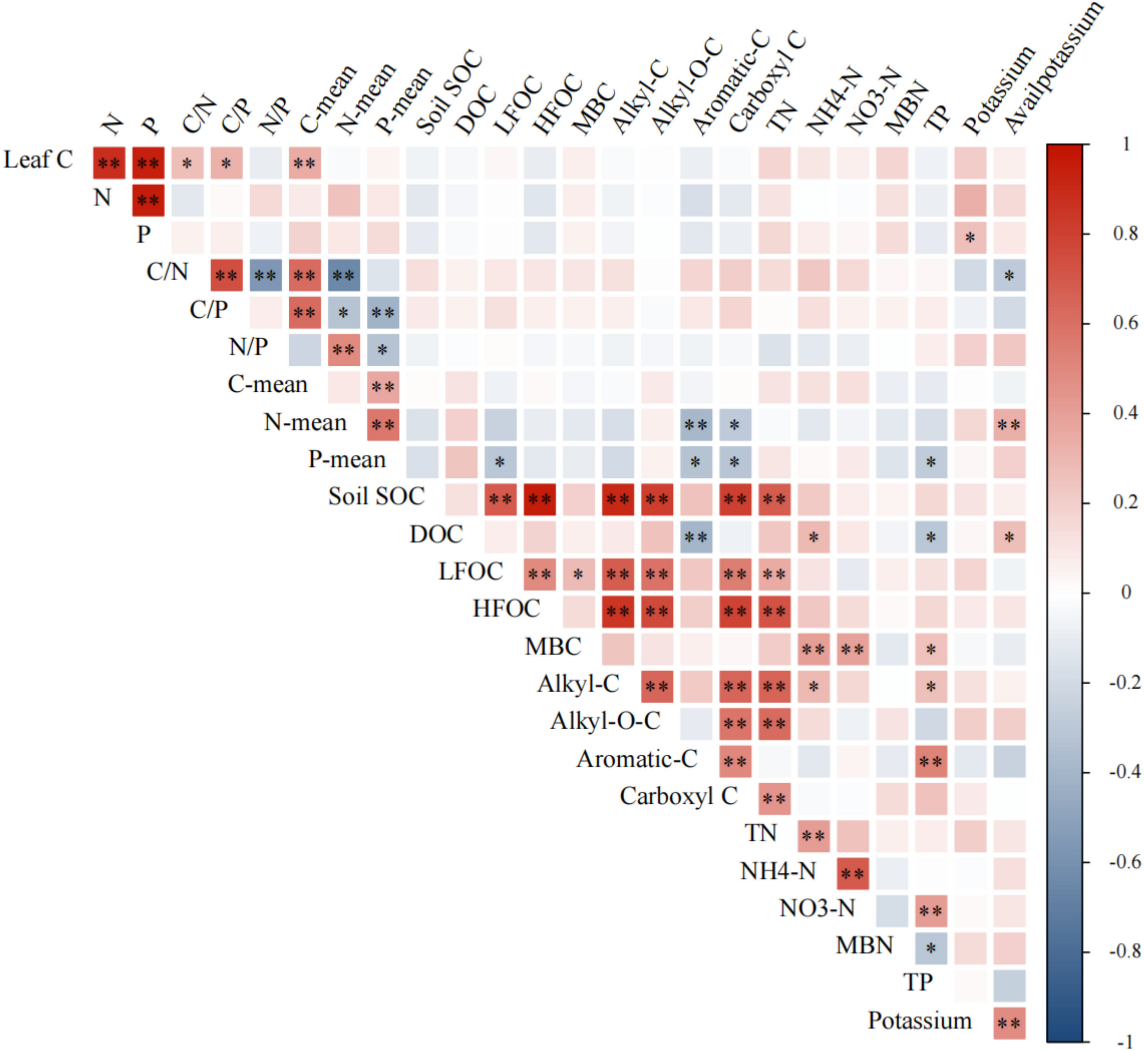
Correlation analysis of leaf litter C, N and P contents and their ratios with soil physicochemical properties. Note: * denotes significance at the 0.05 level (two-sided) and ** indicates significance at the 0.01 level (two-sided).

**Figure 6** shows the correlation analyses for the stoichiometric characteristics of leaf litter and the relationships between C:N, C:P and N:P ratios and soil physicochemical properties in the 60-ha tropical montane rainforest dynamics monitoring plot within Jianfengling. A significant positive correlation was found between the C content in leaf litter and the C:N and C:P ratios. However, no significant correlation was observed between the P and N contents and the C:N, C:P and N:P ratios. Moreover, the mean C content exhibited a significant positive correlation with both C:N and C:P ratios in leaf litter. The mean N content featured a significant negative correlation with C:N and C:P ratios but exhibited a significant positive correlation with the N:P ratio in leaf litter. Conversely, the mean P content had a significant negative correlation with C:P and N:P ratios in leaf litter. Additionally, both the mean C and N contents in leaf litter were significantly positively correlated with the mean P content. However, no significant correlations were observed between C, N, P contents, their stoichiometric ratios in leaf litter and those in the soil. The mean N content in leaf litter exhibited a significant negative correlation with soil aromatic-C and carboxyl-C and a significant positive correlation with soil potassium content. Furthermore, the leaf P content had significant negative correlations with soil light fraction organic carbon (LFOC), aromatic-C, carboxyl-C and TP.

## 4 Discussion

The C, N P contents and their stoichiometric ratios in the leaf litter from the Jianfengling tropical rainforest in Hainan exhibited moderate variability. The C content of leaf litter observed in this study (242.29–378.28 g/kg) (**Table 1**) is lower than the global range reported by Wang et al. (2011) (370.10–568.00 g/kg) and significantly lower than the values for eastern China reported by Ren and Yu (2011) (374.10–646.50 g/kg, with a mean value of 480.1 g/kg) Carbon in leaves is mainly stored in chloroplasts. The lower carbon content observed in the leaf litter from the Jianfengling rainforest may result from microbial decomposition, which breaks down organic matter and releases nutrients. The N and P contents of leaf litter in this study (geometric means of 4.95 and 0.40 g/kg, respectively) (**Table 1**) are lower than the national averages for 753 plant leaves (geometric means of 18.6 g/kg and 1.21 g/kg, respectively) (Han et al., 2005). Additionally, these values are significantly lower than the global averages for N and P contents (geometric means of 17.66 and 1.42 g/kg, respectively) (Elser et al., 2000; Reich and Oleksyn, 2004). This difference may be attributed to the intense plant competition for limited resources in the Jianfengling region and the inherently low nutrient levels, such as nitrogen and phosphorus, in tropical soils (Peguero et al., 2023). Furthermore, the Jianfengling region in Hainan, located in the tropics, exhibits high temperatures and frequent rainfalls throughout the year (Wardle et al., 2004a). The higher temperatures and precipitation may contribute to the leaching of more mobile N (Hui et al., 2005), which may not be effectively absorbed by plants, resulting in lower N content in the plant leaves. The low P content in plant leaves may be due to insufficient P release from the leaf litter (Wu et al., 2023; Xing et al., 2023). Additionally, the low P content may be attributed to the lower soil P content in the study area compared with other global regions (Zhang et al., 2005).

The N:P ratio of leaf litter in this study (11.06) (**Table 1**) is lower than the average value of 14.4 reported for 753 plant leaves in China but higher than the global average of 11.0 (Dynarski et al., 2023; Tian et al., 2024). The amount and rate of litter decomposition significantly affect forest productivity and nutrient cycling efficiency within ecosystems. Plant biodiversity, at both species and genotype levels, plays a crucial role in litter decomposition and nutrient cycling in forest ecosystems (Njoroge et al., 2022). The observed negative correlation between leaf litter N:P values and decomposition rate (Chapin et al., 2002) suggests that the Jianfengling area in Hainan exhibits a faster litterfall decomposition rate than other regions in China. This could be attributed to higher temperatures and humid climate in the area and increased microbial activity and metabolism of decomposers (Zhang et al., 2023b). At leaf litter N:P ratios higher than 14 and P content below 0.22 g/kg, litterfall decomposition is significantly limited by P. Conversely, at N:P ratios below 14, litterfall decomposition is mainly limited by N (Wang et al., 2018), suggesting that litterfall decomposition in the Jianfengling region is mainly limited by N. However, other studies have indicated that the productivity of tropical subtropical forests at low latitudes is often limited by P (Reich and Oleksyn, 2004; Wardle et al., 2004a; Yan et al., 2008). Bui and Henderson (2013) analyzed the ecological stoichiometry of carbon, nitrogen and phosphorus across different vegetation types in Australia. The results revealed that the N:P ratio was significantly limited by nitrogen in temperate regions and by phosphorus in tropical regions. But due to the high soil P content in Hainan Province, which exceeds the national average but remains below the global average (Zhang et al., 2005; Sardans et al., 2021), this is attributed to the lower leaf litter N:P ratios in the study region compared with the national average but higher than the global average and the predominance of N limitation in litterfall decomposition. This study supplements the previous research on leaf litter data and provides a complete dataset from the 60-ha tropical montane rainforest dynamic monitoring plot in Jianfengling. This confirms the unique chemometric characteristics of the Jianfengling rainforest in Hainan. Future research should explore the effects of factors such as dominant species, seasonal variations, and slope exposure to light on ecological stoichiometry.

No significant correlations are observed between the C, N and P contents and their stoichiometric characteristics of leaf litter and those in the soil (**Figure 6**). This suggests that the C, N and P stoichiometric characteristics of leaf litter in Jianfengling have minimal influence on soil physicochemical properties (Zhang et al., 2023a). These findings are consistent with those of Song et al. (2024), who reported that the amount of annual litter does not significantly affect soil organic carbon (SOC) contents. Most studies have indicated that leaf litter cover increases soil organic carbon content, with over 50% of the primary net productivity being returned to the soil through litterfall. This process is the main pathway for recycling plant nutrients into the soil (Wardle et al., 2004b; Hobbie, 2015; Guo et al., 2021; Liu et al., 2022; Yuan et al., 2024). The lack of significant correlations among soil C, N and P stoichiometry may be attributed to non-additive changes resulting from litterfall decomposition (He et al., 2024; Song et al., 2024). In our study, the ley connection between soil and plant leaves involves N and P traits. This observation supports the growth rate hypothesis, which suggests that fast-growing plants require N- and P-rich resources to meet their high demands for RNA and protein synthesis (Sistla and Schimel, 2012), consistent with findings from large-scale global studies (Reich and Oleksyn, 2004). However, this study does not extensively address nutrient efficiency and cycling in plants within the Jianfengling region and serves as a preliminary basis for more detailed future research.

According to **Table 3**, we can see the changes in the leaf litter content of the four species with altitude. *Beilschmiedia brevipaniculata* and *Castanopsis carlesii* decreased and then increased with altitude, *Gironniera subaequalis* and *Altingia obovate* decreased significantly with increasing altitude. The differences among the four species were mainly due to their different ecological requirements. Nitrogen-fixing plants decreased and then increased with increasing altitude, while non-nitrogen-fixing plants decreased significantly with increasing altitude. As the altitude increases, both light intensity and maximum temperature show a decreasing trend (Xu et al., 2020), and sun-loving plants will have a decrease in leaf litter content when the altitude increases, however, *Beilschmiedia brevipaniculata* shows a decreasing and then an increasing trend, and the leaf litter content of the shade-loving plants likewise appears to be decreasing and decreasing and then increasing, which suggests that light and temperature are not decisive factors for their distribution. It seems that nitrogen-fixing plants are more adaptable to changes in altitude and are able to acclimatize to different environments, while non-nitrogen-fixing plants are more sensitive to changes in altitude, resulting in a greater reduction in the amount of leaf litter. Meanwhile the decrease in leaf litter content of *Gironniera subaequalis* and *Altingia obovate* at increasing slopes may be due to erosion and deposition caused by higher slopes, which would result in loss of leaf litter. The relationship between the four plants and convexity was not significant, which may be due to the fact that convexity was not a major factor in the leaf litter content of these plants. Other factors such as climate and soil type may be more important in influencing the leaf litter content of these plants. There was a significant difference in organic carbon content between nitrogen-fixing and non-nitrogen-fixing types of plants, with nitrogen-fixing types having a significantly higher organic carbon content than non-nitrogen-fixing types because of their ability to fix nitrogen, which in turn improves the nutrient level and growth rate of the plants, leading to an increase in organic carbon content (Guo et al., 2023). It indicates that nitrogen limitation plays a decisive factor in this region, influencing plant functional traits which is consistent with the mentioned process of leaf litter decomposition which is mainly limited by nitrogen.

## 5 Conclusion and outlook

This study utilizes sample plots and samples collected from the Jianfengling area in Hainan to investigate the stoichiometric characteristics of C, N and P in the ‘leaf-litterfall-soil’ system and the effects of ecological traits phylogenetic signals on plant functional traits. The findings indicate that the levels of C, N and P in leaf litter are lower in Jianfengling than those observed at both the national and global scales. Moreover, no differences in leaf litter carbon, nitrogen and phosphorus contents are observed among the subplots and dominant plant species. However, the C, N and P contents across different plant groups exhibit greater variability, indicating significant heterogeneity in the samples. In contrast, the data from the subplots feature less variability, indicating significant homogeneity.

Furthermore, significant positive correlations are observed between C and N, C and P, and N and P in leaf litter. The mean contents of C and N are significantly positively correlated with mean P contents. Additionally, mean C values are significantly positively correlated with both C:N and C:P ratios. Conversely, mean P contents are significantly negatively correlated with C:P and N:P ratios. Mean N values exhibit a significant negative correlation with C:N and N:P but feature a significant positive correlation with N:P in leaves. Overall, litter decomposition is mainly limited by elemental N, as indicated by N:P values below 14 in the leaf litter. There were significant phylogenetic signals for three functional traits, namely nitrogen content, altitude and slope, in the Jianfengling 60-ha forest biodiversity dynamic monitoring plot. Light and temperature are not decisive factors affecting plants distribution. Only 31 dominant species or genera were analyzed in this study, and the number of samples may not be sufficient to represent the whole ecosystem; further only the whole year of 2016 was monitored, without considering the long-term trends and spatial variations. In the future, we will continue to monitor the leaf litter in the tropical montane rainforests of the Jianfengling to understand the long-term trends and patterns of change, and to explore the role and mechanism of nitrogen limitation in the decomposition process of leaf litter.

## Data Availability

The raw data supporting the conclusions of this article will be made available by the author, without undue reservation.
